# Widespread nitrous oxide undersaturation in farm waterbodies creates an unexpected greenhouse gas sink

**DOI:** 10.1073/pnas.1820389116

**Published:** 2019-04-29

**Authors:** Jackie R. Webb, Nicole M. Hayes, Gavin L. Simpson, Peter R. Leavitt, Helen M. Baulch, Kerri Finlay

**Affiliations:** ^a^Department of Biology, University of Regina, Regina, SK S4S0A2, Canada;; ^b^College of Biological Sciences, University of Minnesota, St. Paul, MN 55108;; ^c^Institute of Environmental Change and Society, University of Regina, Regina, SK S4S 0A2, Canada;; ^d^Institute for Global Food Security, Queen’s University Belfast, Belfast, Northern Ireland, BT7 1NN, United Kingdom;; ^e^School of Environment and Sustainability, Global Institute for Water Security, University of Saskatchewan, Saskatoon, SK S7N3H5, Canada

**Keywords:** nitrous oxide, agriculture, farm reservoirs, impoundments, greenhouse gases

## Abstract

Nitrogen pollution and global eutrophication are predicted to increase nitrous oxide (N_2_O) emissions from freshwater ecosystems. Surface waters within agricultural landscapes experience the full impact of these pressures and can contribute substantially to total landscape N_2_O emissions. However, N_2_O measurements to date have focused on flowing waters. Small artificial waterbodies remain greatly understudied in the context of agricultural N_2_O emissions. This study provides a regional analysis of N_2_O measurements in small (<0.01 km^2^) artificial reservoirs, of which an estimated 16 million exist globally. We show that 67% of reservoirs were N_2_O sinks (−12 to −2 μmol N_2_O⋅m^−2^⋅d^−1^) in Canada’s largest agricultural area, despite their highly eutrophic status [99 ± 289 µg⋅L^−1^ chlorophyll-a (Chl-*a*)]. Generalized additive models indicated that in situ N_2_O concentrations were strongly and nonlinearly related to stratification strength and dissolved inorganic nitrogen content, with the lowest N_2_O levels under conditions of strong water column stability and high algal biomass. Predicted fluxes from previously published models based on lakes, reservoirs, and agricultural waters overestimated measured fluxes on average by 7- to 33-fold, challenging the widely held view that eutrophic N-enriched waters are sources of N_2_O.

## Significance

Inland water bodies are currently considered to be nitrous oxide (N_2_O) sources to the atmosphere, based on limited studies on large lakes and reservoirs. However, emissions from small artificial waterbodies, such as farm reservoirs, are currently unaccounted for in global models. We present a regional-scale study of N_2_O in farm reservoirs and demonstrate that the majority of these waterbodies act as N_2_O sinks. Our findings contradict previously held assumptions that nitrogen-enriched and eutrophic surface waters within agricultural landscapes are strong sources of N_2_O. The underlying physical and chemical properties driving regional N_2_O consumption may be representative of another 16 million small artificial reservoirs that exist globally, potentially reducing their overall greenhouse gas impact.

Freshwater ecosystems are regarded as globally significant sources of nitrous oxide (N_2_O). Global emissions from rivers are estimated at 0.68 Tg N_2_O-N⋅y^−1^, while lakes and reservoirs contribute an additional ∼0.3 Tg N_2_O-N⋅y^−1^ (1, 2). Combined, inland freshwaters may represent ∼15% of anthropogenic N_2_O emissions, although the global N_2_O budget has yet to include standing water bodies such as lakes, reservoirs, and wetlands (3). Current N_2_O emission estimates from lentic systems are limited by a lack of data, with only 309 global published reports of N_2_O measurements relative to 7,824 and 561 for CO_2_ and CH_4_, respectively (2). Uncertainty in N_2_O levels in lentic systems is further exacerbated by a geographical bias in N_2_O measurements (4), the highly variable nature of freshwater N_2_O fluxes across temporal and spatial scales (5–7), and incomplete understanding on the drivers of N_2_O uptake at the freshwater surface (3).

Net flux of N_2_O from standing waters may be strongly affected by eutrophication, particularly with inorganic nitrogen (N) (8). Some of the highest N_2_O emission rates have been reported in agricultural drainage waters that receive excess N in runoff from manure and crop fertilizers (9–11). Drains, streams, and rivers within agricultural catchments can contribute from 4% to 45% of total landscape N_2_O emissions (12–14), indicative of a potentially important role for other agricultural waters in N_2_O emissions. Critically, landscape mass-balance models predict that nitrous oxide emissions from surface waters will nearly double with the forecast increases in N use and eutrophication (2, 15).

Agricultural landscapes contain large numbers of natural ponds, wetlands, and small constructed waterbodies (hereafter reservoirs). An estimated 16 million small (<0.01 km^2^) artificial reservoirs exist globally (16), yet N_2_O data for these systems are lacking (2.6% of synthesized global lentic N_2_O measurements) (2). Many of these small systems are situated directly in agricultural catchments (17) and have proved to be of critical importance to landscape N cycling. For example, those sized between 0.001 and 0.1 km^2^ represent ∼25% of global lake and reservoir N removal from watersheds (18). Small farm water bodies also have a disproportionate influence on carbon cycling and CO_2_ and CH_4_ emissions (19, 20), yet their contribution to N_2_O emissions remains to be established. Constraining all N_2_O fluxes in agricultural systems is important given that terrestrial N_2_O emissions can offset >100% of CO_2_ uptake associated with enhanced primary production in agriculture (21). Since agricultural reservoirs tend to be N rich and because elevated N influx favors N_2_O production, the omission of agricultural reservoirs may represent a key gap in global N_2_O budgets and greenhouse gas inventories.

To address these shortcomings, we provide an estimate of N_2_O concentrations in small agricultural reservoirs in one of the world’s largest agricultural regions, the Northern Great Plains of North America. Analysis of 101 small artificial waterbodies showed that the majority of farm reservoirs were atmospheric sinks of N_2_O, with 67% of all sites distinctly undersaturated despite mostly eutrophic waters which were rich in dissolved inorganic N. These findings suggest a need to reevaluate our mechanistic understanding of controls on N_2_O in lentic waters and indicate that farm reservoirs may provide a means of minimizing greenhouse gas (GHG) emissions in agricultural grasslands.

## Results and Discussion

Nitrous oxide concentrations spanned three orders of magnitude (1.14–110 nM), with a median of 6.55 nM across all surveyed sites (*SI Appendix*, Table S1). Considering the average uncertainty in N_2_O samples (σ = 1.25 nM), 67% were undersaturated, 21% were supersaturated, and 12% were in relative equilibrium with the atmosphere (∼8.83 nM). These agricultural reservoirs were usually eutrophic, nutrient-rich waterbodies, with concentrations of total dissolved nitrogen (TDN) (417–14,280 µg N⋅L^−1^), dissolved inorganic nitrogen (DIN) (32–7,688 µg N⋅L^−1^), and chlorophyll-*a* (Chl-*a*) (2.2–2,484 µg⋅L^−1^) varying two to three orders of magnitude. Average reservoir TDN (3,082 µg N⋅L^−1^) and phytoplankton abundance (Chl-*a*, 99 µg⋅L^−1^) greatly exceeded global averages (∼800 µg N⋅L^−1^ and ∼20 µg⋅L^−1^, respectively) observed in prior N_2_O studies of lakes and reservoirs (2, 4). As such, these reservoirs represent some of the most nutrient-rich and eutrophic systems where N_2_O measurements have been carried out and challenge the assumption that N-enriched surface waters are potent N_2_O sources (4, 8).

**Fig. 1.**
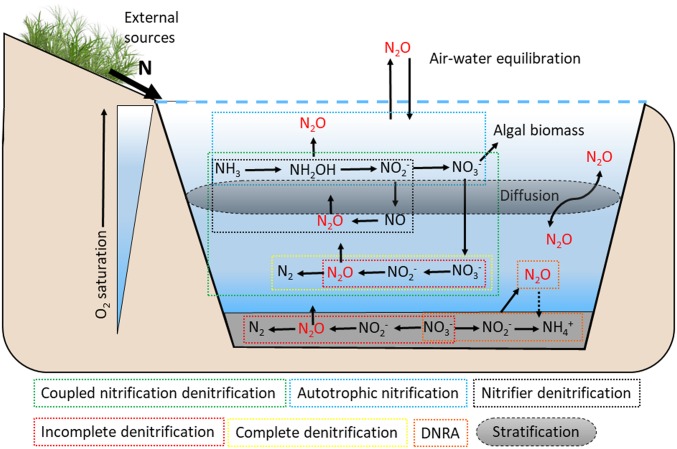
Conceptual diagram of potential N_2_O processes and pathways in agricultural reservoirs. The known physical and microbial processes that influence N_2_O concentrations are depicted by solid arrow lines. The dashed arrow line indicates the potential for N_2_O uptake via DNRA bacteria, although evidence is limited.

Reservoir N_2_O concentrations are the result of complex interactions among potential pathways of N_2_O production and consumption (Fig. 1). To evaluate the potential importance of controls on reservoir N_2_O concentrations, we assessed the relationship between dissolved gas concentrations and common environmental variables known to influence N_2_O. Predictor variables included surface- and bottom-water O_2_ saturation, pH, ratios of TDN to soluble reactive phosphorous (SRP), Chl-*a*, sediment C:N ratio, maximum buoyancy frequency (BF), and DIN concentration. As previous studies have demonstrated that simple linear regressions are often inadequate for predicting freshwater N_2_O concentrations and fluxes (5, 22), we used generalized additive models (GAMs) capable of modeling nonlinear and nonmonotonic relationships to quantitatively assess the effect of environmental conditions and develop a predictive model for our reservoir N_2_O concentrations (*Materials and Methods*).

**Fig. 2.**
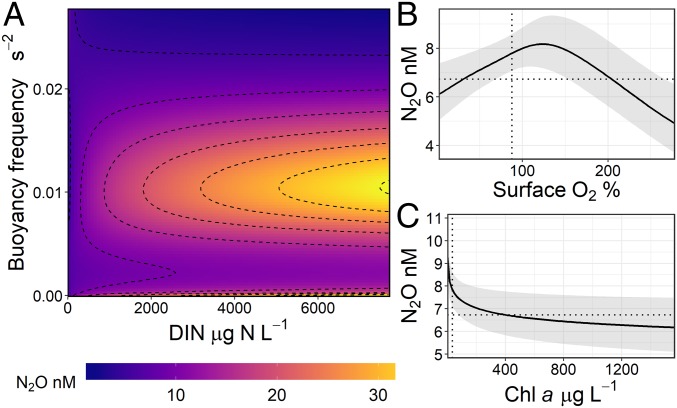
GAM model illustrating partial effect plots for N_2_O concentrations (nM) with significant environmental variables for the agricultural reservoirs. Responses are best explained by (*A*) the interaction between buoyancy frequency and dissolved inorganic nitrogen (DIN), (*B*) surface O_2_, and (*C*) Chl-*a* concentrations. Shaded area in *B* and *C* indicates 95% credible intervals, while dotted lines represent medians of the predictor and response variables. Deviance explained by the model was 85%. Complete statistics can be found in *SI Appendix*, Table S2.

Analysis with GAMs showed that N_2_O concentrations were predicted well by a combination of maximum buoyancy frequency and dissolved inorganic nitrogen (*P* < 0.001), surface dissolved oxygen levels (*P* < 0.05), and Chl-*a* content (*P* < 0.05) but not by other measured parameters (*SI Appendix*, Table S2). The use of a GAM allowed us to detect nonuniform trends in predictor variables, resulting in a model that explained ∼85% of deviance in N_2_O concentrations (*SI Appendix*, Table S2). The lowest N_2_O concentrations occurred under strongly stratified conditions and during periods of high phytoplankton abundance and primary productivity (Fig. 2). Elevated DIN content boosted N_2_O concentrations only when reservoirs were unstratified (BF = 0 s^−2^) or weakly stratified (BF = 0.01 s^−2^) (Fig. 2A).

Our finding demonstrates that elevated DIN content does not invariably result in supersaturation of water-column N_2_O in small reservoirs and depends instead on the degree of water-column stratification. Strong stratification influenced the degree of N_2_O undersaturation across low to high DIN concentrations, with elevated N_2_O levels only in reservoirs that had high DIN and lacked strong stratification (Fig. 2A). This interactive effect suggests that denitrification, a two-step process capable of both producing and consuming N_2_O, is switching between production and consumption below the thermocline (Fig. 1). First, weak stratification may allow for a more spatially variable oxic–anoxic interface in response to diurnal heating and cooling cycles. This enhanced interaction between reduced and oxidized solutes can support coupled nitrification–denitrification under the presence of high DIN (Fig. 1) (23, 24). Second, strong and persistent stratification often promotes N_2_O undersaturation via complete denitrification in hypolimnetic waters that exhibit persistently low O_2_ (23, 25). Alternately, strong stratification may physically limit transfer between the epilimnion and the hypolimnion (25). However, epilimnetic N_2_O consumption is rarely observed in lakes (26) and the precise mechanism remains unknown.

The significant negative response of N_2_O to O_2_ supersaturation and elevated Chl-*a* concentrations may indicate a degree of nutrient competition between primary producers and N_2_O-producing microorganisms in the epilimnion (Fig. 2 B and C) (27). Primary producers assimilate available DIN and are likely limiting any N_2_O production via nitrification, a process that is constrained to the surface layer where oxic conditions prevail (Fig. 1). The negative effect of increasing surface DO above saturation also supports this theory of competition, although the highest levels of DO saturation did not correlate with the highest algal abundance. Dissolved O_2_ can fluctuate greatly over shorter time scales and may represent alternate controlling processes. A diel effect of O_2_ on N_2_O levels has been observed in previous freshwater studies, where lower concentrations coincide with DO peaks (22, 28), although N_2_O remains supersaturated in that research. Thus, the mechanisms linking low N_2_O content to O_2_ supersaturation in our reservoirs remain unexplained and require further research.

**Fig. 3.**
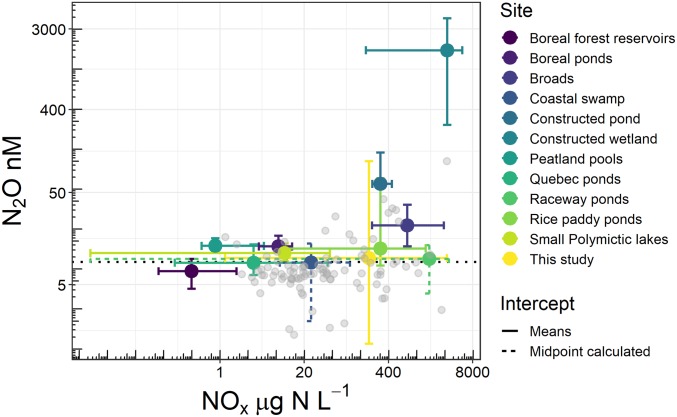
Range of N_2_O and NO_x_ concentrations measured in small lentic systems. Error bars represent upper and lower ranges in each dataset. The concentration of N_2_O at equilibrium is represented by the dotted line. Gray circles are individual reservoirs from our study. See *SI Appendix* for full references.

Evidence of N_2_O consumption has been reported in some other small productive lentic systems and has been attributed to limiting N conditions which support complete denitrification (29, 30). For example, ponds used for microalgae cultivation (29) experience N_2_O undersaturation as NO_x_ becomes limiting (Fig. 3), while small, strongly stratified, hypereutrophic lakes exhibit only trace N_2_O concentrations below the thermocline (30). The observation of strong N_2_O consumption below the stratified layer in the column supports our findings for a primary role of water-column stratification as a regulator of N_2_O processes and concentration gradients. If N_2_O consumption is occurring below the thermocline, then a diffusion gradient of N_2_O between the epilimnion and the hypolimnion can be strong enough to deplete N_2_O in the surface layer (30, 31). Further research into how the physical and biological controls interact to support or limit mechanisms of N_2_O consumption in small, N-enriched waters is required.

Complex interactions between stratification intensity and N content may help explain inconclusive relationships between eutrophication, nutrient status, and N_2_O emissions seen elsewhere (25–28). In other small lentic systems, high N_2_O concentrations have been found to be caused by elevated NO_x_ or NH_4_ concentrations (32), high algal abundance (2, 33), or spatial variation in redox conditions (34). However, some studies report a negative relationship between NO_x_ and N_2_O (35), others suggest either high or low redox conditions support N_2_O production (12, 34, 36), while the presence of algal biomass has been proposed to either enhance or limit N_2_O production in surface waters (29, 34). We believe results of our GAM analysis help resolve these contrasts by identifying the nonlinear relationships between N_2_O concentrations and environmental conditions (Fig. 2). Specifically, we show that physical stratification appears to interact with DIN content to either inhibit or promote N_2_O production, depending on the strength of thermal or chemical stratification.

Our finding that N-enriched small agricultural reservoirs were undersaturated in N_2_O may have broader implications for the N_2_O source or sink behavior of the 16 million other small artificial reservoirs that exist globally (16). Comparing our sites across a limited number of N_2_O measurements made in similar-sized systems, we demonstrate that undersaturatation of N_2_O is not universal in small waterbodies (Fig. 3). Indeed, some of the highest N_2_O concentrations are observed at NO_x_ concentrations >400 µg N⋅L^−1^; however, variability within and between systems for both N_2_O (1–3,930 nM) and NO_x_ (0.1–5,500 µg N⋅L^−1^) is large and no significant relationship exists across all studies (Fig. 3). Interestingly, most of our sites remained undersaturated in N_2_O at NO_x_ ranges that typically support highly supersaturated N_2_O concentrations in other small lentic systems (Fig. 3). This pattern clearly illustrates the need for further work in small ecosystems. Given that our study contains the largest dataset to date on N_2_O measurements in small waterbodies, we provide substantial evidence that not all waterbodies act as N_2_O sources under elevated DIN.

Taking the average of measured reservoir-specific gas transfer velocity (*Materials and Methods*) and extrapolating to all 101 sites, calculated N_2_O fluxes were small (median = −4.03 µmol⋅m^−2^⋅d^−1^), with most acting as sinks rather than large sources of N_2_O (*SI Appendix*, Table S1). For those sites with N_2_O uptake (69 sites), calculated fluxes ranged from −12 to −2 µmol⋅m^−2^⋅d^−1^, whereas sites which were sources (20 sites) varied in strength from 2.21 to 166 µmol⋅m^−2^⋅d^−1^. Fluxes from the remaining sites (12 sites) that were close to atmospheric equilibrium could not be considered as distinct sinks or sources (−1.97 to 1.38 µmol⋅m^−2^⋅d^−1^). Few studies have reported negative N_2_O fluxes in lentic systems (range −60 to −0.08 µmol⋅m^−2^⋅d^−1^), and those that do are often characterized as low N environments (5, 37, 38). In contrast, flowing waters in agricultural catchments tend to act as strong N_2_O sources (93–2,500 µmol⋅m^−2^⋅d^−1^) (12–14), a pattern which is consistent with the observation that agricultural ponds and wetlands have significantly lower N_2_O emissions (three- to ninefold) than flowing waters (12, 36).

While our comprehensive spatial study provides baseline evidence for the role of these systems as N_2_O sinks, research is needed to quantify the temporal variability in N_2_O content in artificial waterbodies among different seasons. It is likely that our late-summer sampling period represents a time when small waterbodies are N_2_O sinks, as both primary productivity and water-column stratification are greatest at this time. Limited studies have assessed the temporal variation of N_2_O concentrations in small artificial waterbodies, yet those that have report conflicting seasons when emissions peak (34–36). This lack of pattern may reflect the differences in regional climate among studies or local variation in physicochemical conditions that regulate O_2_ conditions and inorganic N availability. For example, in the Northern Great Plains, the duration of N_2_O influx is likely governed by the length of stable stratification and autotrophic activity, a period which normally lasts approximately 5 mo (39, 40).

**Fig. 4.**
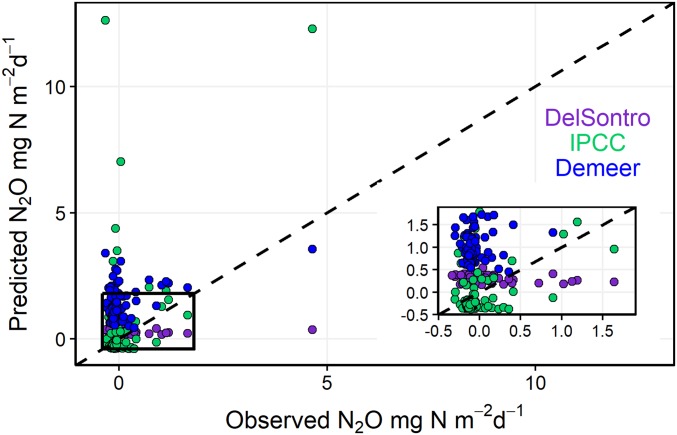
Predicted and observed fluxes for our sites using three different models: (*i*) IPCC N_2_O emission factor (0.0025) for agricultural surface waters based on NO_x_ concentrations (IPCC, 2006), (*ii*) SPW model from DelSontro et al. (2) using lake surface area and trophic status (Chl-*a* concentrations), and (*iii*) strongest model predicting N_2_O fluxes from Deemer et al. (4) using NO_x_ concentrations.

Application of previously published empirical relationships (2, 4, 41) to our reservoirs overestimated potential N_2_O emissions by 7.5- to 33-fold (Fig. 4). Other studies have developed predictive models using water-body area, Chl-*a*, and NO_x_ for global lakes and impoundments (2, 4). Those models often overestimated N_2_O fluxes in our reservoirs when observed fluxes were negative, with differences between calculated and model-predicted values ranging from −0.27 to 13 mg N_2_O-N⋅d^−1^. For example, the Intergovernmental Panel on Climate Change (IPCC) methodology for agricultural GHG inventories predicts N_2_O emissions will increase linearly with NO_x_ concentrations in streams and riverine systems (41). Applying the average 11-fold overestimate of N_2_O fluxes predicted by the IPCC to agricultural reservoirs throughout Saskatchewan produces an estimated 10,530 tonnes (t) CO_2_ equivalent emissions⋅y^−1^, whereas the actual mean measured N_2_O fluxes totaled only 968 t CO_2_ equivalent emissions⋅y^−1^ (42). These observations suggest that global models may not apply universally to similarly designed small artificial waterbodies.

Additional measurements of N_2_O in other agricultural waterbodies are required to further assess their ability to reduce agricultural emissions. Small reservoirs are common in many agricultural regions of the globe, with over 3 million in the United States (43, 44), India (45), Australia (46–48), and Saskatchewan (49) alone, ranging in density from 0.1% to 6% of agricultural land area (17). With a high density of 10 reservoirs on a 1-km^2^ block of farmland in Saskatchewan, agricultural reservoirs have the ability to reduce up to 1–4% of soil N_2_O emissions (0.68–0.95 kg N_2_O-N⋅ha^−1^⋅y^−1^) (50) as CO_2_ equivalents (42) if all were acting as strong sinks. Although the total effect of agricultural reservoirs on integral greenhouse gas emissions is potentially limited by their small landscape area, our key finding remains that these artificial waterbodies do not contribute to significant N_2_O emissions characteristic of other surface waters. For example, if small farm reservoirs in Saskatchewan exhibited N_2_O effluxes as high as other eutrophic systems on farms such as drainage canals (8 mg N_2_O-N⋅m^−2^⋅d^−1^) (12), these basins would account for as much as 66–73% of total farm N_2_O emissions.

In summary, we add an additional 101 sites (∼32%) to the sparse global dataset of lake and reservoir N_2_O measurements. Importantly, this study provided regional-scale insight into the functioning of agricultural water bodies as N_2_O sinks. Given that millions of small agricultural reservoirs exist worldwide, yet have rarely been studied for GHG emissions, we highlight a call to action for further N_2_O measurements in countries with large agricultural areas. This work reveals that in contrast to many lake and reservoir studies, the majority of our agricultural reservoirs act as N_2_O sinks despite having elevated N concentrations, with N_2_O fluxes as much as 33-fold lower than model predictions for lakes and reservoirs in agricultural regions. Nonlinear relationships between stratification, DIN, and N_2_O content reveal the need to reassess the role of small waterbodies as N_2_O sources and consider the environmental controls leading to freshwater N_2_O sinks. Despite their shallow nature and perceived unimportance of vertical structure, stratification in particular should be considered in other small waterbodies when investigating N_2_O dynamics. Inclusion of small reservoirs in agricultural basins may provide an important means of anthropogenic N retention, while providing a potential avenue to reduce net GHG emissions in agriculture.

## Materials and Methods

### Sampling Sites.

A spatial survey of 101 constructed agricultural reservoirs was conducted in a 5-wk period from July to August 2017 across southern Saskatchewan in Canada, covering a total area of 235,000 km^2^ (*SI Appendix*, Fig. S1). Sites were selected from a database of farm reservoirs collected by a survey of regional landowners, as well as from sites on federal lands. Site selection was refined by ensuring a relatively even spatial distribution across the study area, while also considering ease of access. Sites spanned latitudinal and longitudinal ranges of 49° 2′ 25″ to 52° 43′ 9″ and −109° 51′ 6″ to −103° 22′ 42″, respectively. We sampled a diversity of sites that ranged in size from 90 to 13,900 m^2^ and that covered broad land-use types including grassland, pasture, and crop land. The sample area is dominated by intensive agriculture (80%) (51) which contains >110,000 manmade agricultural reservoirs (“dugouts”) (49) to provide onsite surface water storage. The region experiences a subhumid to semiarid climate (Köppen D*fb* classification), with an average annual rainfall ranging from 200 to 500 mm across the prairie ecoregion of the province (52). The cultivated plains are characterized by countless shallow lakes and small pond-like water bodies, most of which are highly evaporative and generally receive most of their surface water from spring snow melt (53). Soils consist of glacial till composed of carbonates derived from limestone bedrock, which leads to the hard-water properties observed in many of the surface waterbodies in the region (39, 54).

### Field Measurements.

A Yellow Springs Instruments (YSI) multiparameter sonde was used to measure water temperature, oxygen concentration, conductivity, salinity, total dissolved solids (TDS), and pH at the surface and at every 0.5 m to just above the sediment surface of each reservoir. Samples for nutrients (nitrogen, NO_3_ + NO_2_, NH_4_, and TDN; and phosphorus, PO_4_ and total P), and Chl-*a* were collected at 0.5 m depth below the surface using a battery-operated pump. These samples were pump filtered through 0.45-µm pore filters in the laboratory on the same day as collection and stored at 4 °C in dark glass bottles until analysis. All nutrients were analyzed following procedures detailed in Leavitt et al. (55). Samples for Chl-*a* were collected on 0.45-mm pore membrane filters and analyzed using spectrophotometry following procedures outlined in Donald et al. (56). Sediment samples were collected to quantify total carbon and nitrogen content in the uppermost 10 cm using an Ekman grab sampler and frozen at −10 °C until analysis. Sediments were freeze dried and ∼2 mg was packed into tin capsules before analysis for carbon and nitrogen content (percentage of dry weight) on an NC2500 Elemental Analyzer (ThermoQuest, CE Instruments).

### Buoyancy Frequency.

The Brunt–Väisälä buoyancy frequency was used as a measure of thermal stratification strength to provide an indication of water column stability. Buoyancy frequency was calculated from temperature profiles based on the density gradient using the package “rLakeAnalyzer” (57) in R (58). We then used the maximum buoyancy frequency for each site in our analysis.

### N_2_O Concentrations and Flux.

Samples for N_2_O were collected at ∼0.3 to 0.5 m below the surface at the deepest point of each reservoir by filling a 1.2-L glass serum bottle using a submersible pump. Atmospheric air was added (60 mL) to the sealed bottle to create a headspace and it was shaken vigorously for 2 min. Two analytical replicates of the equilibrated headspace were extracted with an airtight syringe and dispensed to 12-mL evacuated Exetainer vials with double wadded caps. A sample for atmospheric air was also taken at each site and used to back calculate the original N_2_O concentration in the water. Headspace concentrations were measured using gas chromatography with a Scion 456 Gas Chromatograph (Bruker Ltd.) and calculated using standard curves. Dry N_2_O molar fractions were converted to concentrations as a function of added atmospheric N_2_O concentration, local atmospheric pressure, and N_2_O solubility at the measured water temperature and salinity (59).

Average daily fluxes ($f_{N_{2}O}$) were calculated using water-column N_2_O concentration (*C*_*water*_) and average reservoir gas transfer velocity ($k_{N_{2}O}$) using the equation

$$f_{N_{2}O}=k_{N_{2}O}\left(C_{water}-C_{air}\right),$$ [1]

where $f_{N_{2}O}$ is the flux of N_2_O (mmol ⋅m^−2^⋅d^−1^) and *C*_*ai*r_ is the concentration of N_2_O in the water at atmospheric equilibrium. The average global mixing ratio for the sampling period of 0.33 µatm was used for calculating equilibrium concentrations (Mauna Loa NOAA station, June–August 2017). A series of floating-chamber (0.23 m^2^ area, 0.046 m^3^ total volume) measurements were carried out at a subset of sites (10) to derive a reservoir-specific gas transfer velocity (*k*). Briefly, a total of 30 incubations were taken, with changes in gas concentration recorded by taking five gas samples every 2.5 min for 10 min using a 30-mL syringe, and injected into preevacuated 12-mL vials. The five concentrations were then used to calculate the flux for each incubation using linear regression,

$$f_{gas}=\left(\frac{sV}{mVS}\right)t,$$ [2]

where *s* is the slope of the of the gas change in the chamber (µatm⋅min^−1^), *V* is the volume of the chamber (L), *S* is the chamber surface area (m^2^), *m* is the molar volume of the gas at current atmospheric pressure, and *t* is a conversion factor from minutes to day.

Due to small N_2_O fluxes measured at our chosen sites, a change in concentration was often too low to be detected with confidence over a 10-min incubation period. N_2_O fluxes were determined to be unacceptable due to either the change in chamber N_2_O ppm concentration being within the analysis accuracy (2%) or having an *r*^2^ value <0.7 in the linear regression (60). Instead, the measured CH_4_ flux was used for deriving estimates of *k* due to a stronger detection in the gas accumulation rate. Excluding those incubations that displayed step-like jumps in gas accumulation, which suggests an ebullition event, a total of 23 measurements were used. $k_{CH_{4}}$ (m⋅d^−1^) was calculated using the inverted equation of Fick’s law for gas diffusion,

$$k_{CH_{4}}=\frac{f_{CH_{4}}}{k_{H}\left(p_{CH_{4}water}-p_{CH_{4}air}\right)},$$ [3]

where $f_{CH_{4}}$ is the measured CH_4_ flux (mmol⋅m^−2^⋅d^−1^), *k*_*H*_ is Henry’s constant, and $p_{CH_{4}water}$ and $p_{CH_{4}air}$ are the CH_4_ partial pressures (µatm) in the water and ambient air, respectively. The average global mixing ratio for the sampling period of 1.85 µatm was used for $p_{CH_{4}air}$ (Mauna Loa NOAA station, June–August 2017). All CH_4_ values required for this calculation were collected and analyzed from the same sample as described for N_2_O above. From $k_{CH_{4}},$ the gas transfer velocity was converted to $k_{N_{2}O}$ by using their respective Schmidt numbers ($Sc_{N_{2}O}$ and $Sc_{CH_{4}}$), assuming a Schmidt exponent of 0.67 as follows:

$$k_{N_{2}O}={(\frac{Sc_{N_{2}O}}{Sc_{CH_{4}}})}^{0.67}k_{CH_{4}}.$$ [4]

The average $k_{N_{2}O}$ calculated from the floating-chamber incubations was 1.64 ± 1.24 m⋅d^−1^.

The original data generated in this study are freely available on GitHub and Zenodo (61).

### Statistical Methods.

#### Variable selection.

To assess the predictability of environmental drivers on controlling N_2_O concentrations, we selected a range of variables known to affect N_2_O production and consumption pathways. Fig. 1 provides an overview of the assumed processes potentially influencing N_2_O in our artificial agricultural reservoirs. We included both surface and bottom water dissolved oxygen (DO) saturation. Dissolved oxygen represents the extent of oxic and anoxic conditions in the water column, which has been shown to exert strong controls over consumption and production processes including nitrification, incomplete and complete denitrification, and dissimilatory nitrate reduction to ammonium (DNRA) in aquatic environments (62). Sediment C:N molar ratio was used as a proxy for carbon substrate availability, which is a key energy source to heterotrophic communities such as denitrifiers and those facilitating DNRA (63). Chlorophyll-*a* was included as a measure of phytoplankton abundance and trophic status (2). The amount of production by algal biomass can serve as an indicator for N loading and, if especially abundant, may compete for inorganic carbon and nitrogen substrates in the upper water column (Fig. 1). Reservoir pH (measured at the surface) was included as low pH has been shown to limit nitrification rates and stimulate N_2_O sink behavior (5, 64). TDN to SRP ratio (mg:mg^−1^) was included to provide a measure of N limitation, as well as the concentration of inorganic nitrogen species (DIN = NH_4_ + NO_x_) that are directly involved in all N_2_O processes (Fig. 1). Finally, Brunt–Väisälä buoyancy frequency was included as a physical control for surface water and bottom water mixing. Physical stratification can promote the development and persistence of anoxic conditions in the hypolimnion, allowing more opportunity for N_2_O consumption to persist throughout the water column (23, 26).

#### Modeling procedures.

To test our hypotheses, we used a GAM to estimate the effects of covariates on reservoir N_2_O concentrations. We anticipated nonlinear relationships between covariates and N_2_O concentrations, and GAMs allow for the estimation of smooth, nonlinear relationships without requiring the functional form of the relationship to be specified. Instead, the estimated effects are learned from the data themselves.

To guard against multicollinearity issues before model fitting, the Pearson product moment correlation coefficient was estimated for all pairs of candidate predictor variables selected to reflect processes shown in Fig. 1. Pairs of variables that were highly correlated with one another were screened, and the ones most directly related to our hypotheses and the processes shown in Fig. 1 were retained. Chl-*a*, N:P, and DIN were log-transformed (base *e*), while BF was square-root transformed to achieve better dispersion of observed variables. The GAM estimated was

$$N_{2}O∼Gamma\left(\mu ,\theta \right)$$ [5]

$$g\left(\mu_{i}\right)=\alpha +f_{1}\left(SurfDO_{i}\right)+f_{2}\left(DeepDO_{i}\right)+f_{3}\left(SedCN_{i}\right)+f_{4}\left(\text{log}\left(Chla_{i}\right)\right)+f_{5}\left(SurfpH_{i}\right)+f_{6}\left(\text{log}\left(N:P_{i}\right)\right)+f_{7}\left({\sqrt{BF}}_{i},\text{log}\left(DIN\right)\right)+\gamma_{i},$$

where the conditional distribution of N_2_O concentration is assumed Gamma distributed with expected value ***μ*** and other parameters ***θ***. Value ***μ*** was modeled as a linear combination of smooth functions (*f*_*k*_) of *k* covariates for the *i*th sample. Smooth *f*_7_ is a bivariate tensor product smooth representing the marginal effects and interaction of BF and DIN, produced from marginal cubic regression spline bases each composed of four basis functions. The remaining smooths used univariate thin plate regression spline bases each comprising nine basis functions. A random reservoir effect was included as a random intercept (*γ*_*j*_) for the *j*th reservoir. *g*() is the log link function.

Quadratic penalties on the integrated squared second derivatives of each spline were used to determine the complexity of estimated smooth functions. These penalties operate on the range space of each penalty and as such cannot be used to shrink individual terms out of the model. To achieve model selection therefore, we included a second penalty on the null space (the perfectly smooth components of a set of basis functions) of each penalty, using the “double penalty” approach (65). In combination, the two types of penalty per smoother allow for terms that are not related to the response to be effectively removed from the model, a process known as shrinkage or regularization.

All model coefficients and penalties were estimated using restricted marginal likelihood (65, 66) with the *mgcv* package (67) for R (58). A summary of the model results is provided in *SI Appendix*, Table S2.

### Literature Model Comparison.

Observed N_2_O fluxes were compared with three different models in the literature. For IPCC predictions of indirect N_2_O emissions from agricultural drainage waters, fluxes were calculated using the emission factor assigned to surface waters of 0.0025 and known concentration of NO_x_, referred to as the EF(B) method (56). This ratio was derived from studies on streams, rivers, groundwater, and estuaries, as currently no emission factor estimate is available for lentic systems (41). Fluxes were then calculated by taking the predicted N_2_O concentration and our average reservoir gas transfer velocity ($k_{{\text{N}}_{2}\text{O}}$) following Eq. **1**. The second model applied used surface area and Chl-*a* as predictor variables from a linear model of global lake and impoundment N_2_O flux measurements (2). The third model compared used NO_x_ only from a linear model derived from a global database of constructed reservoir N_2_O flux measurements (18). All predicted fluxes were converted to mg N⋅m^−2^⋅d^−1^ for comparison.

## Supplementary Material

Supplementary File
