# Supplementary material for: Widespread nitrous oxide undersaturation in farm waterbodies creates an unexpected greenhouse gas sink
Source: Proc Natl Acad Sci U S A. 2019 Apr 29;116(20):9814–9. doi: 10.1073/pnas.1820389116 (PMC6525509; doi:10.1073/pnas.1820389116)
Supplement: Supplementary File [file pnas.1820389116.sapp.pdf]

PNAS
www.pnas.org

Supplementary Information for

Widespread nitrous oxide undersaturation in farm waterbodies creates an unexpected greenhouse gas sink

J. R. Webb[1]*, N.M. Hayes[1,2], G. L. Simpson[1,3], P.R. Leavitt[1,3,4], H.M. Baulch[5], and K. Finlay[1]

[1]Department of Biology, University of Regina, Regina, SK, S4S0A2, Canada.
[2]College of Biological Sciences, University of Minnesota, Twin Cities, MN 55108, USA
[3]Institute of Environmental Change and Society, University of Regina, Regina, Saskatchewan, Canada, S4S 0A2
[4]Institute of Global Food Security, Queen's University Belfast, Belfast, Northern Ireland, United Kingdom.
[5]School of Environment and Sustainability, Global Institute for Water Security, University of Saskatchewan, 11 Innovation Boulevard, Saskatoon, SK S7N3H5, Canada

*J. R. Webb
Email: jackie.roslyn.webb@gmail.com

www.pnas.org/cgi/doi/10.1073/pnas.1820389116

This PDF file includes:

Figs. S1
Tables S1 to S3

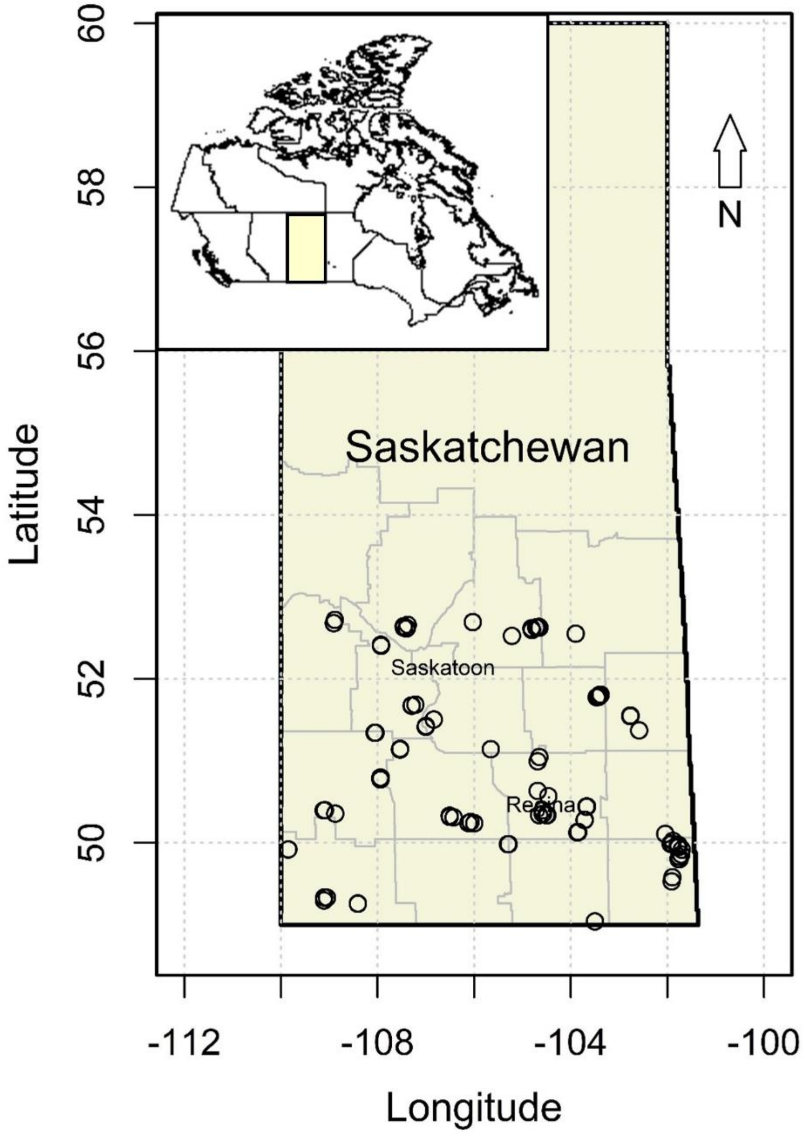

SI Fig. S1. Map of sampled reservoir locations in the prairie region of Saskatchewan, Canada.

SI Table S1: Summary statistics of nitrous oxide concentrations, reservoir chemistry, and physical attributes.

**Parameter**	**Units**	**Mean**	**Median (minimum and maximum)**	***n***
Area	m^2	1,336	1,080 (90-13,900)	98
Depth	m	2.11	2.10 (0.18-5.10)	100
Surface temperature	°C	20.10	19.90 (15.70-29.50)	101
Bottom temperature	°C	16.90	17.10 (6.4-22.6)	98
Surface DO	%	91.76	87.70 (2.30-344)	101
Bottom DO	%	25.89	3.00 (0-128)	97
pH		8.74	8.74 (6.95-10.19)	101
Salinity	ppt	0.87	0.45 (0.07-8.57)	101
Buoyancy frequency	s^{-2}	0.007	0.004 (0.00-0.028)	97
N_2O	nM	9.71	6.55 (1.14-110)	101
N_2O flux	µmol m^{-2} d^{-1}	1.48	-4.03 (-12.33-174)	101
NH_3	µg N L^{-1}	360	100 (10-5,930)	98
NO_3+NO_2	µg N L^{-1}	200	34.12 (1.21-3,189)	97
DIN	µg N L^{-1}	557	137 (31.83-7,688)	97
TDN	µg N L^{-1}	3,082	2,360 (417-14,280)	97
Ch- a	µg L^{-1}	99.05	36.88 (2.23-2,484)	101
TP	µg P L^{-1}	284	80.00 (8.70-6,480)	97
N:P	mg : mg^{-1}	180	57.54 (1.59-8,661)	96
Sediment C:N	mol : mol	15.21	13.97 (6.50-31.84)	98

SI Table S2. Statistical summary of GAM model used to assess multivariate drivers of N_2O nM. Predictor variables included were surface dissolved oxygen saturation (*SurfDO*), bottom dissolved oxygen saturation (*DeepDO*), sediment C:N ratio (*SedCN*), chlorophyll a (*Chla*), surface pH (*SurfpH*), water column N:P ratio (*N:P*), maximum buoyancy frequency (*BF*), and dissolved inorganic nitrogen (*DIN*), with reservoir site included as a random intercept (γ_j).

Model term	Ref edf	edf	F statistic	*p*-value
f(SurfDOi)	1.9	9	5.4	0.02*
f($DeepDO_i$)	1.6E-4	9	0.0	0.72
f($SedCN_i$)	4.2E-4	9	0.0	0.38
f(log(Chlai))	0.79	9	3.1	0.03*
f($SurfpH_i$)	1.7E-4	9	0.0	0.87
f(log(N:Pi))	2.3E-4	9	0.0	0.49
f(sqrt(BFi), log(DIN_i))	7.3	24	21.8	1.2E-9***
Random intercept (γ_j)	51	87	1.8	3.14E-10***
**Intercept coefficient:** 2.05 ± 0.05				
**Adjusted r^2:** 0.69				
**Deviance explained:** 85.3%				

Significance codes *0.05, **0.01, ***0.001

SI Table S3: References in Figure 3

**Boreal forest reservoirs**	Hendzel LL, Matthews CJD, Venkiteswaran JJ, St. Louis VL, Burton D, Joyce EM, Bodaly RA. 2005. Nitrous Oxide Fluxes in Three Experimental Boreal Forest Reservoirs. Environmental Science & Technology 39: 4353-4360.
**Boreal ponds**	Huttunen JT, Väisänen TS, Heikkinen M, Hellsten S, Nykänen H, Nenonen O, Martikainen PJ. 2002. Exchange of CO_2, CH_4 and N_2O between the atmosphere and two northern boreal ponds with catchments dominated by peatlands or forests. Plant and Soil 242: 137-146.
**Broads**	Outram FN, Hiscock KM. 2012. Indirect Nitrous Oxide Emissions from Surface Water Bodies in a Lowland Arable Catchment: A Significant Contribution to Agricultural Greenhouse Gas Budgets? Environmental Science & Technology 46: 8156-8163.
**Constructed pond**	Gao Y, Zhang Z, Liu X, Yi N, Zhang L, Song W, Wang Y, Mazumder A, Yan S. 2016. Seasonal and diurnal dynamics of physicochemical parameters and gas production in vertical water column of a eutrophic pond. Ecological Engineering 87: 313-323.
**Coastal swamp**	Ueda S, Go C-S, Yoshioka T, Yoshida N, Naohiro, Wada E, Miyajima T, Sugimoto A, Boontanon N, Vijarnsorn P, Boonprakub S. 2000. Dynamics of dissolved O_2, CO_2, CH_4, and N_2O in a tropical coastal swamp in southern Thailand. Biogeochemistry 49: 191-215.
**Constructed wetland**	Søvik AK, Kløve B. 2007. Emission of N_2O and CH_4 from a constructed wetland in southeastern Norway. Science of The Total Environment 380: 28-37.
**Peatland pools**	Arsenault J, Talbot J, Moore TR. 2018. Environmental controls of C, N and P biogeochemistry in peatland pools. Science of The Total Environment 631-632: 714-722.
**Quebec ponds**	Soued C, del Giorgio PA, Maranger R. 2015. Nitrous oxide sinks and emissions in boreal aquatic networks in Québec. Nature Geoscience 9: 116.

**Raceway ponds**	Ferrón S, Ho DT, Johnson ZI, Huntley ME. 2012. Air–Water Fluxes of N_2O and CH_4 during Microalgae (Staurosira sp.) Cultivation in an Open Raceway Pond. Environmental Science & Technology 46: 10842-10848.
**Rice paddy ponds**	Xia Y, Li Y, Ti C, Li X, Zhao Y, Yan X. 2013. Is indirect N_2O emission a significant contributor to the agricultural greenhouse gas budget? A case study of a rice paddy-dominated agricultural watershed in eastern China. Atmospheric Environment 77: 943-950.
**Small polymictic lakes**	Whitfield CJ, Aherne J, Baulch HM. 2011. Controls on greenhouse gas concentrations in polymictic headwater lakes in Ireland. Science of The Total Environment 410: 217-225.
